# Preparation and characterization of Acanthopanax polysaccharides nanoselenium with enhanced stability and antioxidant activity

**DOI:** 10.3389/fnut.2025.1712826

**Published:** 2025-11-19

**Authors:** Yichao Feng, Xueping Zhang, Yichen Ge, Chenglin Li, Yaosen Yang, Jianqing Su, Xiuling Chu

**Affiliations:** College of Agriculture and Biology, Liaocheng University, Liaocheng, China

**Keywords:** *Acanthopanax senticosus* polysaccharide, nanoselenium, characterization, antioxidant activity, stability

## Abstract

To address the issue of nanoselenium easily aggregating and becoming inactive, *Acanthopanax senticosus* polysaccharides were used as a stabilizer to construct *Acanthopanax senticosus* polysaccharide nanoselenium for potential nutritional applications. ASP-SeNPs were synthesized using a chemical reduction method with ultrasonic assistance, and response surface methodology was used to optimize the preparation conditions to control particle size. The characterization results show that the basic structure of polysaccharides in ASP-SeNPs is retained, and they are mainly bound to nanoselenium through hydrogen bonds or coordination bonds. ASP-SeNPs particle size distribution ranged from 58 to 123 nm, with an average zeta potential of −27.8 mV. A one-month stability test showed that ASP-SeNPs had superior stability compared to conventional SeNPs, remaining stable for 30 days at 4 °C and for 20 days at room temperature (25 °C). Furthermore, ASP-SeNPs exhibited free radical scavenging activity against DPPH (1,1-diphenyl-2-picryl-hydrazyl radical), ABTS [2,2′-Azinobis-(3-ethylbenzthiazoline-6-sulphonate)], and hydroxyl radicals. Their IC₅₀ values were, respectively, 76.789 μg/mL, 74.927 μg/mL, 343.419 μg/mL.

## 1 Introduction

Selenium (Se) is an essential trace element crucial for human health, primarily due to its role as a cofactor in antioxidant enzymes such as glutathione peroxidases, which help maintain intracellular redox homeostasis. Imbalances in oxidative and antioxidant systems can lead to oxidative stress, where excessive free radicals damage DNA, proteins, and lipids, contributing to various pathological conditions (1). Consequently, maintaining the optimal selenium status represents a fundamental strategy for preserving redox balance. However, selenium deficiency remains a widespread public health issue, affecting over 50% of the Chinese population due to insufficient daily intake (2). While selenium supplementation is a common strategy to address this nutritional gap, the narrow range between the effective dose and the toxic dose of selenium (the recommended daily intake is 60 μg, with a safe upper limit of 400 μg per day), as excessive intake may precipitate selenosis (3). This highlights the urgent need for novel selenium forms that combine high bioavailability with low toxicity.

Currently available selenium supplements, including both inorganic and organic forms, are often associated with notable toxicity and limited bioavailability (4). In recent years, selenium nanoparticles (SeNPs) have emerged as a promising alternative, offering higher biocompatibility, stronger bioactivity, and reduced toxicity (5). Despite their advantages, SeNPs are still not the most suitable selenium source due to high surface energy and colloidal instability, which often result in particle aggregation and consequent loss of bioactivity (6). To address these limitations, macromolecular stabilizers are typically employed; polysaccharides serve as effective soft templates for controlling SeNP size and morphology due to their complex branched architectures and abundant hydroxyl groups (7) primarily through the widely adopted chemical reduction method (8). Meanwhile, ultrasonic treatment can effectively prevent particle aggregation and promote uniform dispersion, thereby reducing particle size, making it a suitable auxiliary method for the preparation of polysaccharide nanoselenium (9). Extensive research has demonstrated that polysaccharide-encapsulated SeNPs exhibit diverse functional properties. For example, Xu and colleagues developed porphyra haitanensis polysaccharide-functionalized SeNPs that showed significant efficacy in ameliorating ulcerative colitis (10). Similarly, Zhang et al. reported that *Lycium barbarum* polysaccharide-stabilized SeNPs enhanced intestinal selenium absorption (11).

*Acanthopanax senticosus* polysaccharide (ASP), a major bioactive component extracted from the traditional Chinese medicinal herb *Acanthopanax senticosus* (AS), modulates the intestinal microbiota and exhibits antioxidant effects, antimicrobial properties, and anti-inflammatory effects (12). Its strong hydrophilicity and simple extraction process further make it a practical and sustainable candidate for nanoparticle stabilization (13). Building on these advantages, we propose the use of ASP as a novel stabilizer for the green synthesis of functional SeNPs.

This study utilized ASP as a stabilizer, sodium selenite as a selenium source, and Vitamin C (Vc) as a reducing agent to prepare *Acanthopanax senticosus* polysaccharide nanoselenium (ASP-SeNPs) via an ultrasonic-assisted chemical reduction method. The preparation process was optimized through response surface experiments to enhance its colloidal stability and investigate its physicochemical properties and biological activity. The results confirmed that ASP-SeNPs are a new type of selenium source with significant potential for use in nutritional supplements and functional foods aimed at addressing selenium deficiency.

## 2 Materials and methods

### 2.1 Materials and instruments

*Acanthopanax senticosus*, produced in Bozhou, Anhui Province; sodium selenite (CP), purchased from Shanghai Adamas Reagents Co., Ltd.; Vitamin C (AR), purchased from Shanghai McLean Biochemical Technology Co., Ltd.; and DPPH and ABTS, purchased from Sinopharm Chemical Reagent Co., Ltd.

An ultrasonic water bath was used (Ningbo Xinzhi Biotechnology Co., Ltd.) at a power output of 840 W.

### 2.2 Experimental methods

#### 2.2.1 Extraction and purification of ASP

Ultrasound-assisted hot water extraction was used to extract ASP, which was then purified. Protein removal was performed via the Sevage method: Mix chloroform and n-butanol in a 4:1 ratio. Add a solution of Siberian ginseng polysaccharides at three times the volume. Shake for 30 min, then pour into a separatory funnel and retain the supernatant, and decolorization was performed via the hydrogen peroxide decolorization method. Finally, the solution was poured into a dialysis bag (MW 500), dialyzed for 24 h in a flowing water environment, then placed in a vacuum freeze dryer and freeze-dried into a powder.

#### 2.2.2 Preparation of ASP-SeNPs

The redox method was synthesized via prepare ASP-SeNPs, modified from the procedure described by Zu-Man et al. (14) and incorporating an ultrasonic-assisted reaction process. First, Na₂SeO₃ solution and ASP solution were mixed, followed by the addition of Vc solution, under continuous stirring. In this system, the volume ratio of Na₂SeO₃ solution, ASP solution, and Vc solution was 1:1:2. The reaction mixture was placed in an ultrasonic water bath for a thorough reaction and then freeze-dried into powder.

#### 2.2.3 Study of the optimal preparation conditions for ASP-SeNPs

The effects of various reaction conditions (addition of Na₂SeO₃, Vc: Na₂SeO₃, ultrasonic power, temperature, time, and concentration of ASP) on the stability of the ASP-SeNP composites were investigated via a response surface test. Using the double-wavelength colorimetric method for colloidal solutions, the ratio of absorbance at 410 nm to that at 490 nm (A_410_/A_490_) of the prepared ASP-SeNP samples was used as the evaluation criterion. A higher ratio of absorbance values at the two wavelengths indicates a smaller particle size and greater stability of the nanoselenium (15).

##### 2.2.3.1 Single-factor experiment, Plackett–Burman experiment and steepest ascent experiment

Single-factor preliminary experiments were first conducted with five levels for each parameter to systematically evaluate their individual effects on the stability of ASP-SeNPs. Based on the results of the single-factor experiments, a Plackett–Burman experimental design was subsequently employed to screen for key influencing factors, with each parameter set at two levels (high and low). Through this design, three factors demonstrating statistically significant effects were identified. According to both the magnitude and direction (positive or negative) of each factor’s effect observed in the Plackett–Burman experiment, the steepest ascent experiment was used to determine the optimal ranges for the most influential parameters.

##### 2.2.3.2 Box–Behnken experiment

Based on the results of the above experiments, the ultrasonic power was fixed at 40%, the temperature was 60 °C, and the time was 120 min. The addition of Na_2_SeO_3_, Vc: Na_2_SeO_3_, and the concentration of ASP were set as independent variables. A_410_/A_490_ was set as the response value. In accordance with the Box–Behnken experimental principle, a response surface optimization experiment based on three factors and three levels was designed via Design Expert 13.0 software. The factors and level codes of the experiment are shown in Table 1. Each experiment will be conducted in triplicate, and the average value will be taken.

**Table 1:** Factors and levels of Box–Behnken tests.

Level	Factors		
	Addition of Na_2_SO_3_ (mmol/L)	Vc: Na_2_SO_3_	Concentration of ASP (mg/mL)
−1	6	2:1	3
0	5	2.5:1	3.5
1	4	3:1	4

#### 2.2.4 Characterization of ASP-SeNPs

The morphological characteristics of ASP-SeNPs were observed by scanning electron microscopy (SEM, TESCAN MIRA LMS) and transmission electron microscopy (TEM, JEOL JEM-F200). The surface elemental composition of the ASP-SeNPs was analyzed via energy dispersive spectrometry (EDS, TESCAN MIRA LMS). The crystal structure of the ASP-SeNPs was analyzed via X-ray diffraction (XRD, SmartLab). The structural characteristics of the ASP-SeNPs were analyzed via Fourier transform infrared (FT-IR, Thermofisher Scientific Nicolet is10) spectroscopy. The particle size and zeta potential of the ASP-SeNPs and SeNPs were determined via a zeta potential analyzer (DLS, Malvern Zetasizer Nano ZS90).

#### 2.2.5 Stability assessment of ASP-SeNPs

The prepared SeNP solution and ASP-SeNP solution, both containing 3% selenium, were stored at 4 and 25 °C, respectively, in the dark for 30 days. During this period, the ratio of the absorbance at wavelengths of 410 and 490 nm was measured every 5 days, and the color and state of the solution were recorded; this was used as a reference to assess the stability of the ASP-SeNP solution.

#### 2.2.6 *In vitro* antioxidant activity assay of ASP-SeNPs

The *in vitro* antioxidant activity of ASP-SeNPs was evaluated by assessing DPPH radical scavenging, ABTS radical scavenging, and hydroxyl radical scavenging experiments in accordance with methods described previously (16). In these assays, Vc at equivalent mass concentrations was used as the positive control. The half-maximal inhibitory concentration (IC_50_) was subsequently calculated based on the experimental results to quantitatively compare the antioxidant capacity.

#### 2.2.7 Data processing

Data analysis and graphing were performed via software such as Excel 2021, IBM SPSS Statistics 21, Design Expert 13.0, and Origin 2021.

## 3 Results and discussion

### 3.1 Results and analysis of the single-factor experiment

#### 3.1.1 Effect of the addition of Na_2_SeO_3_

**Figure 1:**
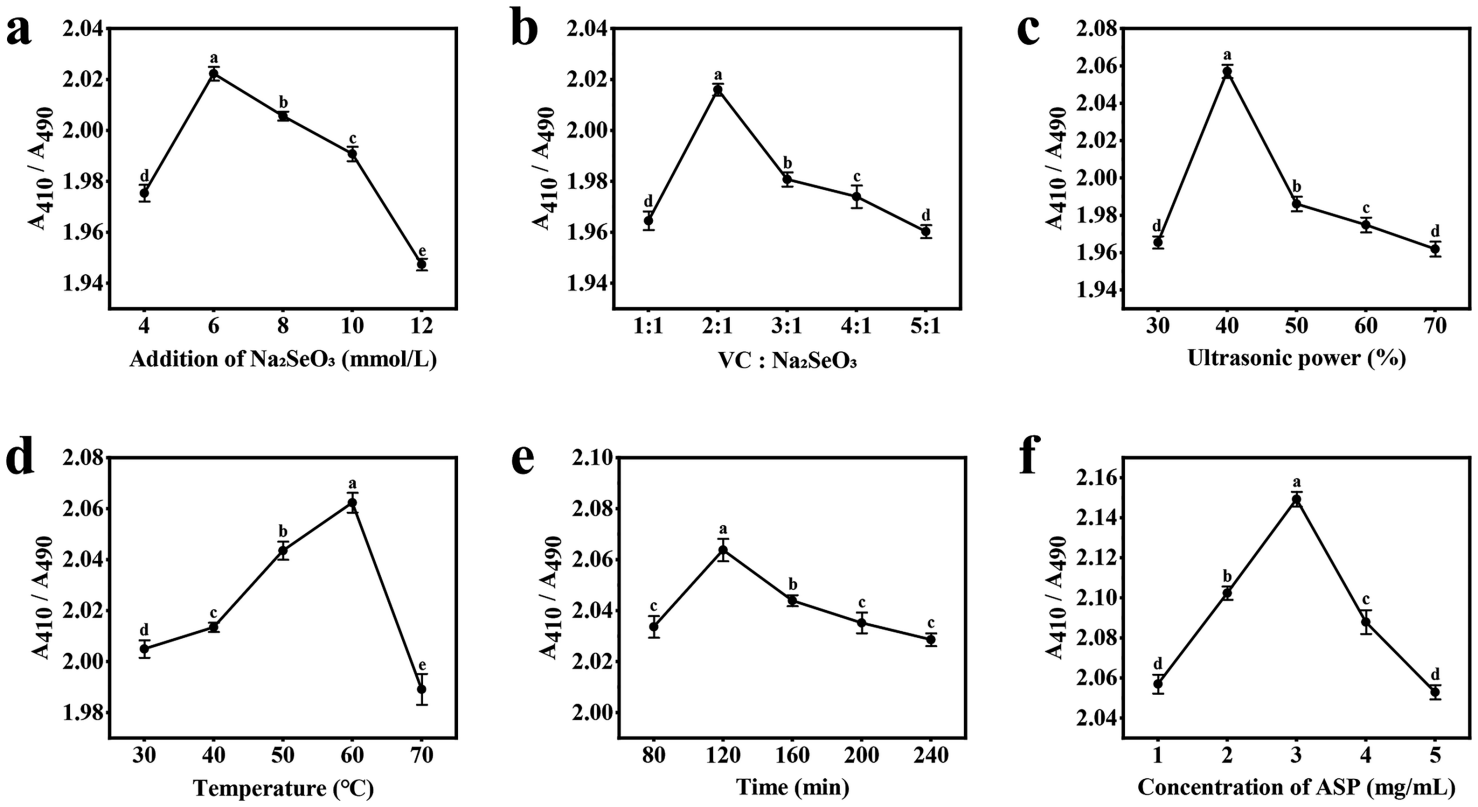
Results of the single-factor experiment. **(a)** Addition of Na2SeO3; **(b)** Vc:Na2SeO3; **(c)** Ultrasonic power; **(d)** Temperature; **(e)** Time; **(f)** Concentration of ASP.

As illustrated in Figure 1a, A_410_/A_490_ exhibited a distinct parabolic trend in response to increasing sodium selenite concentration. The ratio reached its maximum value at an optimal sodium selenite concentration of 4 mM, beyond which a progressive decline was observed. This phenomenon can be attributed to competitive binding effects at higher selenite concentrations. Specifically, excess selenite ions likely saturate the available hydroxyl (-OH) and amino (-NH₂) functional groups on the polysaccharide backbone (17). The observed decrease in A_410_/A_490_ at higher selenite concentrations suggests impaired nanoparticle formation. Under these conditions, unbound selenium atoms may undergo uncontrolled aggregation rather than forming stable complexes with the polysaccharide matrix; this results in larger, less uniform nanoparticles with compromised colloidal stability, as reflected in the altered absorbance characteristics.

#### 3.1.2 Effect of Vc: Na_2_SeO_3_

As shown in Figure 1b, after the addition amount of sodium selenite was determined, the addition amount of Vc was gradually increased, and the value of A_410_/A_490_ first increased before the ratio of the addition amount reached 2:1; this occurred because when Vc is added, the selenium precursor SeO₃^2−^ reacts with Vc in a reduction reaction, resulting in the formation of the more stable polysaccharide nanoselenium (18). However, as the ratio of the two additives increased from 2:1 to 5:1, the A_410_/A_490_ value gradually decreased and stabilized. This occurred because further increasing the Vc concentration led to instability in the reaction system. The excessive reducing agent instantly generated an extremely high concentration of zero-valent selenium atoms. The limited amount of polysaccharide was insufficient to effectively cap and stabilize all of these newly formed nuclei and atoms, which led to increased particle size through aggregation and uncontrolled growth (19).

#### 3.1.3 Effect of ultrasonic power

As illustrated in Figure 1c, when the ultrasonic power was set to 30–40%, the A_410_/A_490_ ratio of the ASP-SeNPs increased, reaching a maximum at 40%, indicating that ultrasonic treatment not only improved the dispersion of nanoselenium particles but also significantly reduced the particle size, which is consistent with the findings of Cai et al. (20). However, the A_410_/A_490_ ratio decreases when the ultrasonic power is set to 50%. When the ultrasonic power is subsequently set to 60–70%, the A_410_/A_490_ tends to stabilize; this may be because high-frequency ultrasonic waves convert acoustic energy into thermal energy, causing the solution to heat. Ultrasonic treatment at different temperatures can lead to changes in particle size, thereby affecting the stability of polysaccharide nanoselenium particles (21).

#### 3.1.4 Effect of temperature

As shown in Figure 1d, the A_410_/A_490_ value of ASP-SeNPs increased with rising water bath temperature during the initial reaction stage. When the reaction temperature reached 60 °C, the ratio reached its maximum, after which it decreased significantly as the temperature continued to rise. The reason for this reaction is that heating causes intense movement of the nanoparticles, increasing the frequency and intensity of collisions between particles; within an appropriate temperature range, this promotes the binding of polysaccharides with nanoselenium, but beyond this range, it exacerbates aggregation, thereby compromising the stability of the polysaccharide nanoselenium (22).

#### 3.1.5 Effect of time

As illustrated in Figure 1e, during the initial stage of the reaction, the A_410_/A_490_ value of the ASP-SeNPs increases with prolonged ultrasonic water bath exposure, reaching a maximum of 120 min. As the reaction time continues to increase, this value begins to decrease; this occurred because as the reaction time increased, the reactions between Na_2_SeO_3_, VC, and ASP became more complete, resulting in more uniformly dispersed ASP-SeNPs. However, if the reaction time is excessively prolonged, it may lead to aggregation of SeNPs in the system, thereby affecting the average particle size (19).

#### 3.1.6 Effect of the concentration of ASP

As shown in Figure 1f, during the early stage of the reaction, as the concentration of the Acanthopanax polysaccharide solution increased, the A_410_/A_490_ value of the ASP-SeNPs gradually increased. This finding is due to the strong physical adsorption of the -OH groups, causing the SeNPs to be adsorbed by the ASP macromolecules, resulting in the formation of more stable ASP-SeNPs. Additionally, the increased soluble polysaccharide content in the system enhances the inhibition of new SeNP aggregation and SeNP adsorption, enabling SeNPs to maintain a stable smaller size (23). When the polysaccharide concentration reached 3 mg/mL, the A_410_/A_490_ ratio reached its maximum value. However, as the polysaccharide concentration continues to increase beyond this point, the A410/A490 ratio begins to decrease continuously; this may be because excessively high polysaccharide concentrations result in more polysaccharide chains binding to the SeNPs on their surfaces, leading to larger hydrated particle sizes (24, 25).

### 3.2 Results and analysis of the Plackett–Burman experiment

The Plackett–Burman experimental design and results are presented in Supplementary Table S1. Variance analysis and significance testing of these results are summarized in Table 2. The model’s *F* value = 9.03 > 1, *p* value = 0.0144 < 0.05, *R*^2^ = 0.9155, and *R*^2^_adj_ = 0.8141, indicating that the model is statistically significant and well-fitted. Based on the *p* value, the influence of the six factors on A_410_/A_490_ is ranked as follows: F (Concentration of ASP) > A (Addition of Na_2_SeO_3_) > B (Vc: Na_2_SeO_3_) > D (Temperature) > C (Ultrasonic power) > E (Time). Among these, A, B and F significantly affected A_410_/A_490_ (*p* < 0.05).

**Table 2:** Statistical analysis of the Plackett–Burman test.

Source	Sum of squares	df	Mean square	*F*-value	*P*-value	Significance
Model	0.0215	6	0.0036	9.03	0.0144	Significant
A	0.0074	1	0.0074	18.53	0.0077	Significant
B	0.0028	1	0.0028	6.92	0.0465	Significant
C	0.0007	1	0.0007	1.68	0.2511	
D	0.0008	1	0.0008	2.01	0.2158	
E	0.0004	1	0.0004	1.03	0.3571	
F	0.0095	1	0.0095	24.01	0.0045	Significant
Residual	0.002	5	0.0004			
Cor total	0.0235	11				

*R*^2^ = 0.9155, *R*^2^_adj_ = 0.8141.

A regression analysis was performed on the data in Supplementary Table S1, yielding the multiple linear regression equation for the six factors and the response value A_410_/A_490_: Y = 2.05–0.0124A + 0.0151B−0.0075C−0.0082D−0.0058E + 0.0282F. Among the significant factors, B and F have positive effects, indicating that as their values increase, the A_410_/A_490_ value tends to increase; A has a negative effect, meaning that as its value increases, the A_410_/A_490_ value tends to decrease.

### 3.3 Results and analysis of the steepest ascent experiment

Considering the synthesis efficiency of ASP-SeNPs and cost control during the preparation process, in the steepest ascent experiment, the four nonsignificant factors were set as fixed values, i.e., ultrasonic power at 40%, temperature at 60 °C, and time at 120 min. The three significant factors determine the direction of the slope based on the positive or negative coefficients in the equation mentioned in section 3.2. The step size is designed based on the results of the single-factor test.

Table 3 shows the design and results of the steepest slope test. As shown in the table, A_410_/A_490_ is the highest in the 5th test group. Therefore, the test conditions from the 5th test are selected as the 0 level in the Box–Behnken experiment.

**Table 3:** Design and results of the steepest ascent experiment.

No.	Addition of Na_2_SeO_3_ mmol/L	VC: Na_2_SeO_3_	Concentration of ASP mg/mL	A410/A490
1	6	2:1	3	2.150813936
2	5.6	2.2:1	3.2	2.178217096
3	5.2	2.4:1	3.4	2.185678706
4	4.8	2.6:1	3.6	2.206379887
5	4.4	2.8:1	3.8	2.249118949
6	4	3:1	4	2.229316578

### 3.4 Results and analysis of the response surface experiment

Based on the results of the Plackett–Burman experiment and the steepest ascent experiment, under fixed conditions of ultrasonic power at 40%, temperature at 60 °C, and time at 120 min, the following response factors were selected: addition of Na_2_SeO_3_ (A), Vc: Na_2_SeO_3_ (B), concentration of ASP (C) as response factors, and A_410_/A_490_ as response values. A three-factor, three-level design with 17 experimental groups was established based on the Box–Behnken experimental design principle. The response surface optimization experimental design and response value results are shown in Supplementary Table S2, and the analysis of variance results is shown in Table 4.

**Table 4:** Analysis of variance of the regression equation for the Box–Behnken test.

Source	Sum of squares	df	Mean square	*F*-value	*P*-value	Significance
Model	0.001	9	0.0001	5.43	0.0182	Significant
A	2.38 × 10–6	1	2.38 × 10–6	0.1186	0.7407	
B	0.0001	1	0.0001	3.97	0.0864	
C	0.0002	1	0.0002	9.93	0.0161	Significant
AB	2.57 × 10–6	1	2.57 × 10–6	0.1282	0.7309	
AC	0.0001	1	0.0001	4.12	0.0819	
BC	1.10 × 10–7	1	1.10 × 10–7	0.0055	0.9431	
A^2^	0.0001	1	0.0001	5.12	0.0582	
B^2^	0.0003	1	0.0003	16.66	0.0047	Significant
C^2^	0.0001	1	0.0001	5.87	0.0459	Significant
Residual	0.0001	7	0			
Lack of fit	0.0001	3	0	0.8599	0.5306	Not significant
Pure error	0.0001	4	0			
Cor total	0.0011	16				

*R*^2^ = 0.8747, *R*^2^_adj_ = 0.7135.

Design-Expert 13.0 software was used to perform regression analysis on the experimental data in Table 4, yielding the following fitted equation: Y = 2.20–0.0005A + 0.0032B + 0.0050C−0.0008AB + 0.0045 AC−0.0002 BC−0.0049A^2^–0.0089B^2^–0.0053C^2^.

As shown by the results of the variance analysis of the response surface in Table 4, the Model *F* value is 5.43, and the *p* value is 0.0182 < 0.05, indicating that the model is significant. The residual term of the model is 0.5306 (*p* > 0.05), indicating that the residual term is not significant; this suggests that the model has good fitting ability and can predict the A_410_/A_490_ of ASP-SeNPs within the range of experimental variables. The coefficient of determination of the model *R*^2^ = 0.8747, and the adjusted coefficient of determination *R*^2^_adj_ = 0.7135, indicating that the model can explain 71.35% of the variation in the A_410_/A_490_ of ASP-SeNPs. The coefficient of variation (CV) indicates the precision of the experiment; the lower the CV value is, the greater the reliability of the experiment. In this experiment, CV = 0.2042%, indicating that the experimental operation is reliable and that the results are trustworthy (26). This model can be used for analyzing and predicting the optimal process for preparing ASP-SeNPs.

### 3.5 Analysis of the response surface experiment’s 3D graph

**Figure 2:**
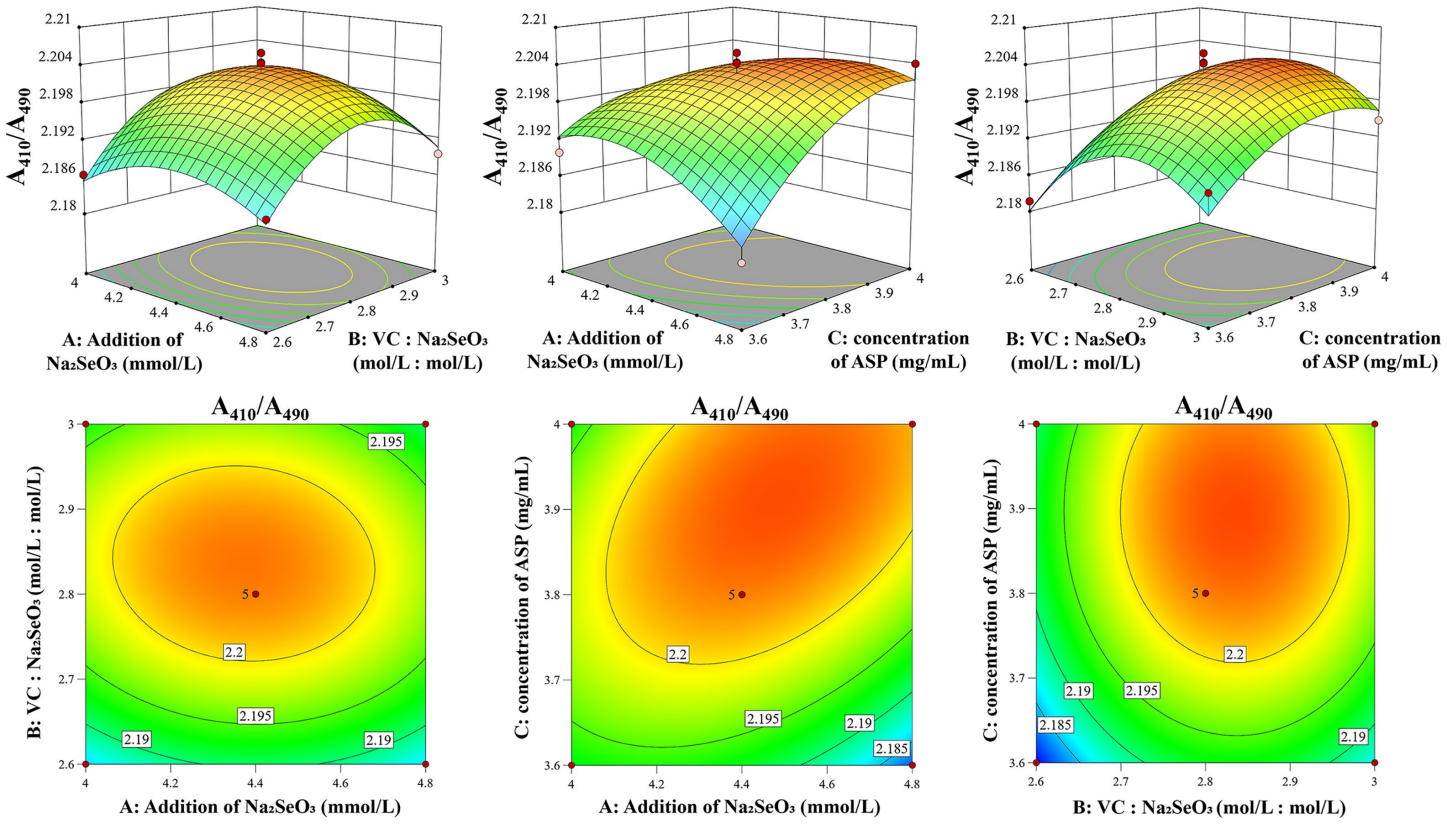
Response surface diagram of the effects of factor interactions on the A410/A490 of ASP-SeNPs.

The morphological characteristics of the contour lines and the gradient of curvature in the response surface three-dimensional plot serve as critical indicators for evaluating interaction effects. The degree of ellipticity and contour density directly correlate with the interaction strength, where more pronounced elliptical patterns and denser contours signify stronger factor interactions. Similarly, steeper curvatures in the response surface topography demonstrate more substantial impacts of factor interactions on the A_410_/A_490_ of ASP-SeNPs (27). As shown in Figure 2, the optimal condition within the experimental domain corresponds to both the response surface’s maximum elevation point and the focal center of the most compact elliptical contour. A comparison of the steepness trends of various factors, reveals that the concentration of ASP (C) has a greater impact on A_410_/A_490_, followed by the Vc: Na_2_SeO_3_ (B) and the addition of Na_2_SeO_3_ (A). The statistical analysis presented in Table 2 reveals that the two-factor interactions among the three investigated variables did not achieve statistical significance (*p* > 0.05), suggesting that the primary effects dominated the response characteristics under the current experimental conditions.

### 3.6 Prediction and validation of optimal preparation conditions

Based on the Box–Behnken experiments, the optimal preparation conditions for ASP-SeNPs were as follows: under a fixed ultrasonic power of 40%, a temperature of 60 °C, and a time of 120 min, the optimal conditions were as follows: the addition of Na_2_SeO_3_ at 4.477 mM, the Vc: Na_2_SeO_3_ at 2.832:1, and the concentration of ASP at 3.909 mg/mL. Under these conditions, the model predicted an A_410_/A_490_ ratio of 2.204 for ASP-SeNPs. Considering practical feasibility in actual operations, the conditions were adjusted to the following: the addition of Na_2_SeO_3_ was 4.48 mM, the Vc: Na_2_SeO_3_ was 2.83:1, the concentration of ASP was 3.91 mg/mL, and the other conditions remained unchanged. Three parallel validation experiments were conducted under the adjusted conditions, yielding an A_410_/A_490_ value of 2.205945, 2.194036, 2.191485 (The average value is 2.1971 ± 0.0077), which is close to the theoretical predicted value, indicating that the model is accurate and has high practical operability.

### 3.7 Results and analysis of characterization

#### 3.7.1 Results and analysis of SEM

**Figure 3:**
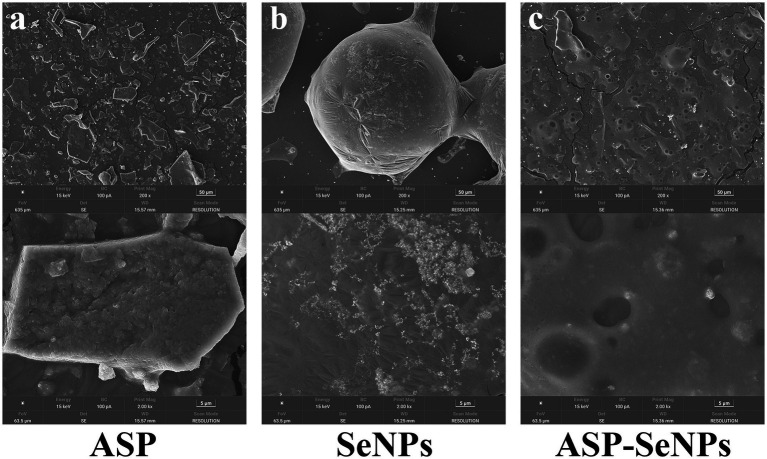
SEM images of ASP **(a)**, SeNPs **(b)**, and ASP-SeNPs **(c)**.

Figure 3 presents representative SEM images comparing the morphological characteristics of ASP (a), SeNPs (b), and ASP-SeNPs (c). The ASP matrix has a characteristically rough and irregular surface topography, featuring numerous binding sites that facilitate nanoparticle attachment. A comparative analysis of the data in Figures 3b,c reveals distinct differences in nanoparticle morphology and distribution. SeNPs demonstrate significant aggregation tendencies and heterogeneous particle shapes, whereas ASP-SeNPs display markedly improved dispersion and uniformity. This morphological transition confirms the effective stabilization role of ASP, where the surface functional groups of the polysaccharide interact with SeNPs to prevent aggregation and promote homogeneous distribution. The observed increase in nanoparticle dispersion underscores the critical role of polysaccharide stabilizers in modulating the physicochemical properties of SeNPs.

#### 3.7.2 Results and analysis of TEM

**Figure 4:**
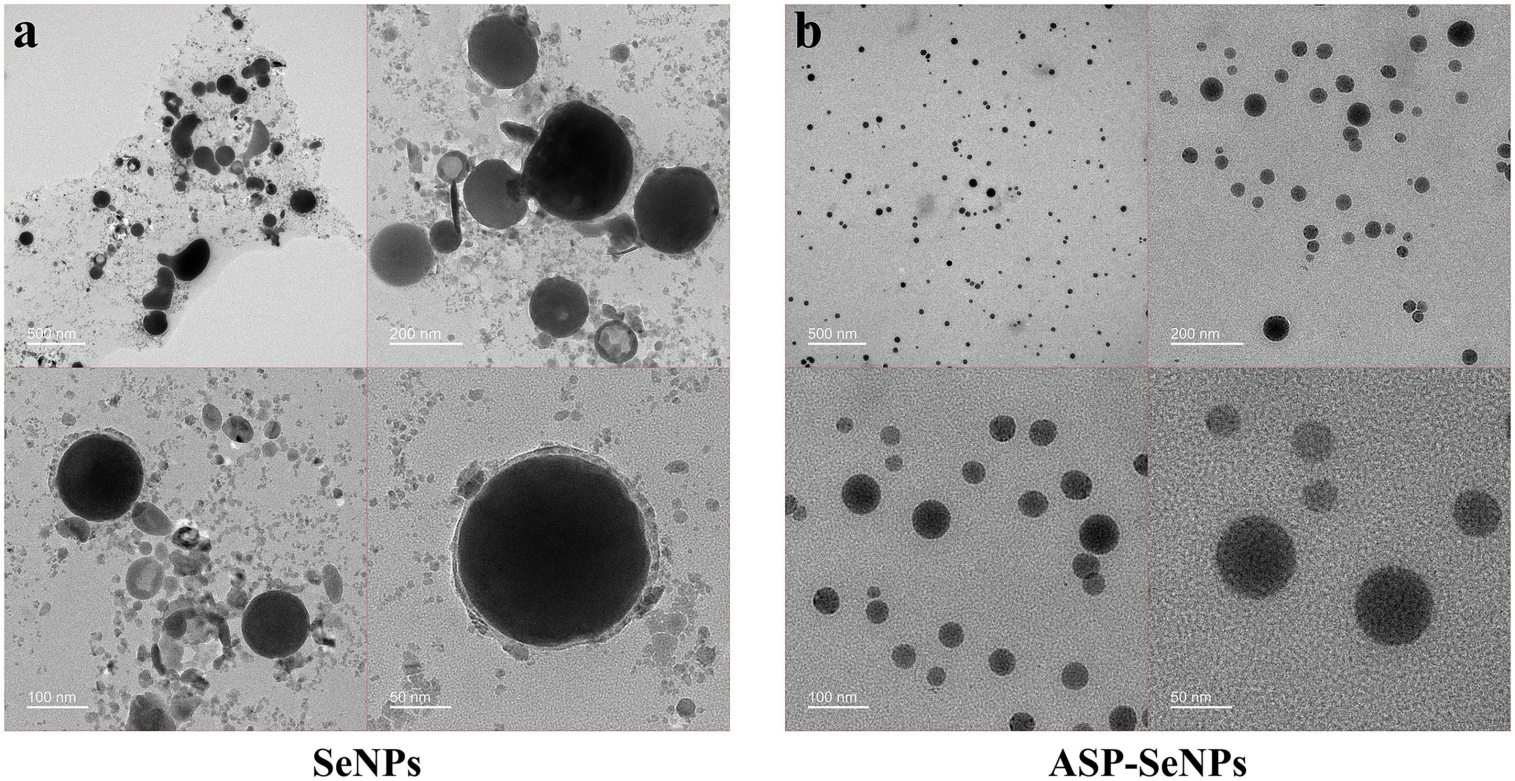
TEM images of SeNPs **(a)** and ASP-SeNPs **(b)**.

Transmission electron microscopy (TEM) analysis revealed distinct morphological differences between the unstabilized and ASP-stabilized SeNPs. The unstabilized SeNPs (Figure 4a) display a heterogeneous particle size distribution with substantial aggregation and poor colloidal stability. In marked contrast, the ASP-SeNP composite (Figure 4b) demonstrated a homogeneous spherical morphology with significantly improved dispersion characteristics, in agreement with previous reports (28). These observations confirmed the successful surface modification of SeNPs by ASP through polysaccharide–nanoparticle interactions, which effectively prevented particle agglomeration. Compared with their unstabilized counterparts, the resulting ASP-SeNPs exhibit enhanced colloidal stability and more uniform nanoscale dimensions, demonstrating the critical role of polysaccharide modification in optimizing the physicochemical properties of SeNPs for use as additives in selenium-enriched foods.

#### 3.7.3 Results and analysis of EDS

**Figure 5:**
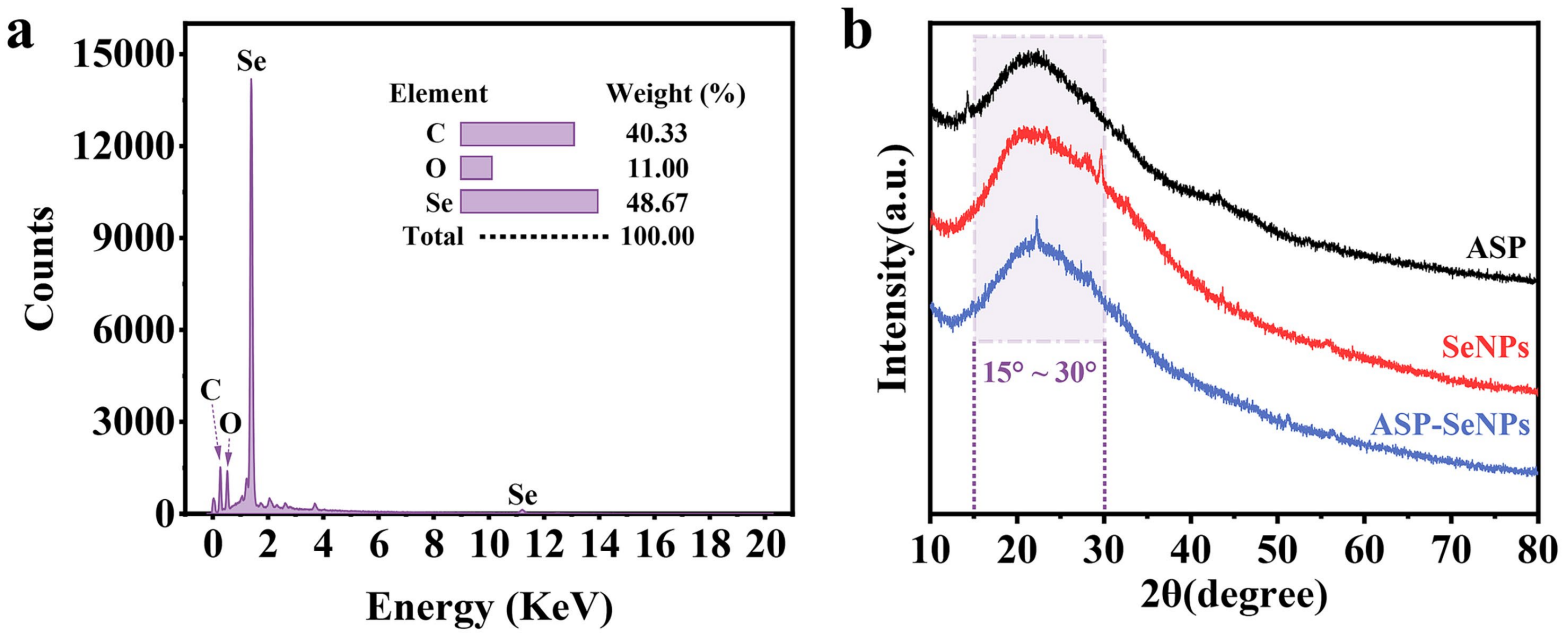
Elemental analysis spectrum of ASP-SeNPs **(a)** and X-ray diffraction patterns of ASP, SeNPs and ASP-SeNPs **(b)**.

The surface elements of the ASP-SeNPs were analyzed, and the results are shown in Figure 5a. The elemental composition of the prepared APS-SeNPs was as follows: C (40.33%), O (11.00%), and Se (48.67%). The spectrum showed strong C and O absorption peaks and relatively strong Se absorption peaks, confirming that ASP and SeNPs were successfully combined and bonded on the surface (19).

#### 3.7.4 Results and analysis of XRD

The intensity and sharpness of X-ray diffraction peaks can to some extent reflect the crystalline nature of a sample. Therefore, XRD was further used to characterize the formation of ASP-SeNPs. As shown in Figure 5b, the X-ray diffraction pattern of ASP exhibited a broad diffraction peak in the range of 15–30°, indicating that ASP has an amorphous structure, which is consistent with the results of other studies on plant polysaccharides (29, 30). The X-ray diffraction patterns of SeNPs and ASP-SeNPs both exhibit broad diffraction peaks at lower angles, characteristic of amorphous phases, which are consistent with the XRD patterns of amorphous selenium reported in the literature. These findings indicate that the prepared SeNPs and ASP-SeNPs are both amorphous selenium. Additionally, the peak shapes of ASP-SeNPs are similar to those of SeNPs and ASP, but their diffraction intensities and peak positions have changed, indicating that ASP and SeNPs have combined to form ASP-SeNPs. Since polysaccharides form hydrogen bonds with nanoselenium through their surface hydroxyl groups (31), the chemical structures of ASP and SeNPs remain unchanged.

#### 3.7.5 Results and analysis of FT-IR

**Figure 6:**
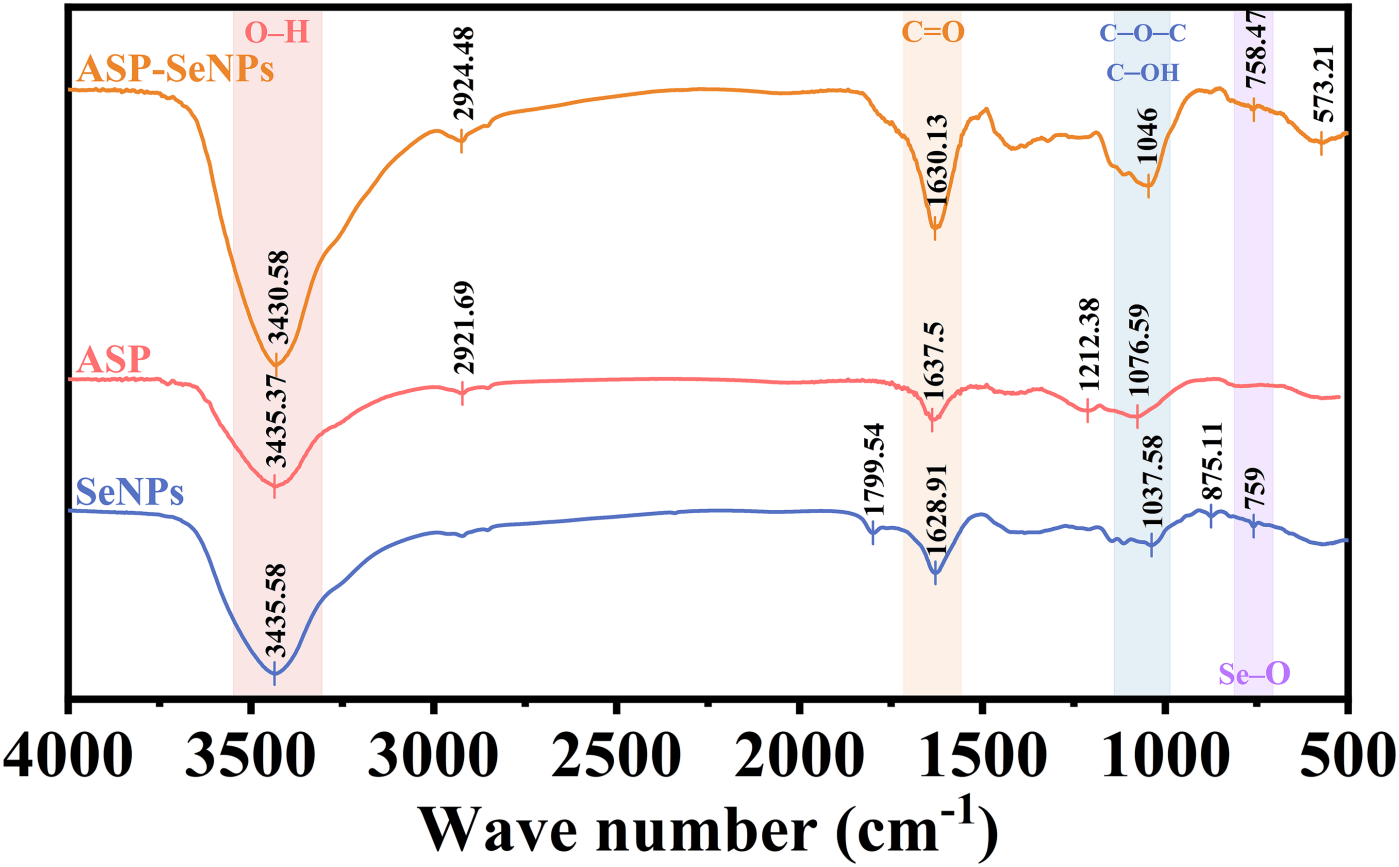
FT-IR diagrams of SeNPs, ASP and ASP-SeNPs.

Fourier transform infrared spectroscopy can perform qualitative and quantitative analyses of material structures by measuring the absorption or reflection characteristics of samples (32). Figure 6 shows the Fourier transform infrared spectra of ASP-SeNPs, ASP, and SeNPs. As shown in the figure, ASP exhibited a broad peak at 3,435 cm − 1, which corresponds to the O-H stretching vibration peak of polysaccharides. The weak peak at 2,922 cm-1 is attributed to the C-H stretching vibration peak. Indicating the CH₂ or CH₃ groups of sugar rings or side chains. The peak at 1,637 cm^−1^ may correspond to the bending vibration of adsorbed water (H-O-H) or a small amount of C=O (carboxyl group). The peak at 1,077 cm^−1^ corresponds to the stretching vibrations of C-O-C (glycosidic bond) and C-OH (primary alcohol), which are characteristic signals of the polysaccharide backbone (33). The infrared spectrum of ASP-SeNPs retains the characteristic peaks of polysaccharides (such as O-H and C-O-C), but the O-H stretching peak in ASP-SeNPs shows a slight redshift compared with that in ASP, and the transmittance is lower, indicating a higher O-H content or stronger vibrational activity. At the same time, ASP-SeNPs also exhibit new peaks in the low wavenumber region (at 573 and 758 cm^−1^), which may correspond to Se-O bonds or Se-C bonds. These results suggest that no new covalent bonds are formed between ASP and SeNPs; rather, they are bound via hydrogen bonds or coordination bonds (24).

#### 3.7.6 Results and analysis of particle size and the zeta potential

**Figure 7:**
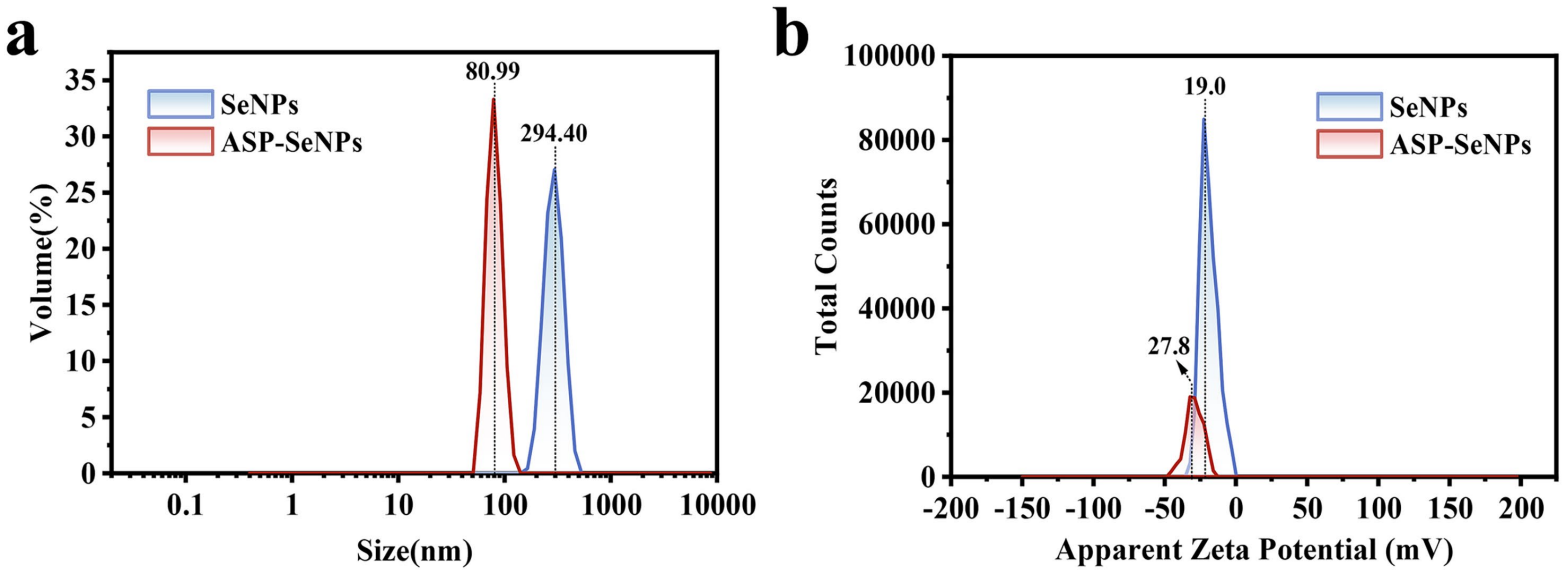
Particle size distributions of SeNPs **(a)** and ASP-SeNPs and zeta potential distributions of SeNPs and ASP-SeNPs **(b)**.

The particle size distribution diagrams of SeNPs and ASP-SeNPs are shown in Figure 7a. The particle size distribution of the SeNPs ranged from 164 to 459 nm, with relatively low and broad volume peaks, indicating a wide particle size distribution range, possible aggregation, and poor stability. In contrast, the particle size distribution of ASP-SeNPs primarily falls within the range of 58 to 123 nm. The average particle sizes of polysaccharide-based SeNPs prepared by Gao et al. (34) and Chen et al. (24) also fall within this range. Additionally, their peak volumes are relatively higher and narrower, indicating a more uniform particle size distribution, fewer aggregates, and a more stable system. These results demonstrate that, compared with traditional SeNPs, ASP-SeNPs synthesized with ASP as a stabilizer have smaller particle sizes and greater stability.

The surface charge characteristics were analyzed through zeta potential measurements (Figure 7b), revealing that both SeNPs and ASP-SeNPs exhibit negative zeta potentials, indicating that both types of nanoparticles carry negative charges on their surfaces, which is consistent with the findings of several previous studies (24). Among these zeta potentials, the average potential of SeNPs (−19.0 mV) is closer to zero than that of ASP-SeNPs (−27.8 mV), indicating that the absolute value of the zeta potential for the latter is greater than that for the former. These findings indicate that, compared with SeNPs, ASP-SeNPs have stronger electrostatic repulsion, making the particles less prone to aggregation and increasing the stability of the dispersion system. Combining the above analysis of particle size, the zeta potential results not only further confirm that ASP-SeNPs are more stable than traditional SeNPs but also serve as one of the reasons for this phenomenon.

### 3.8 Results and analysis of the stability experiment

**Figure 8:**
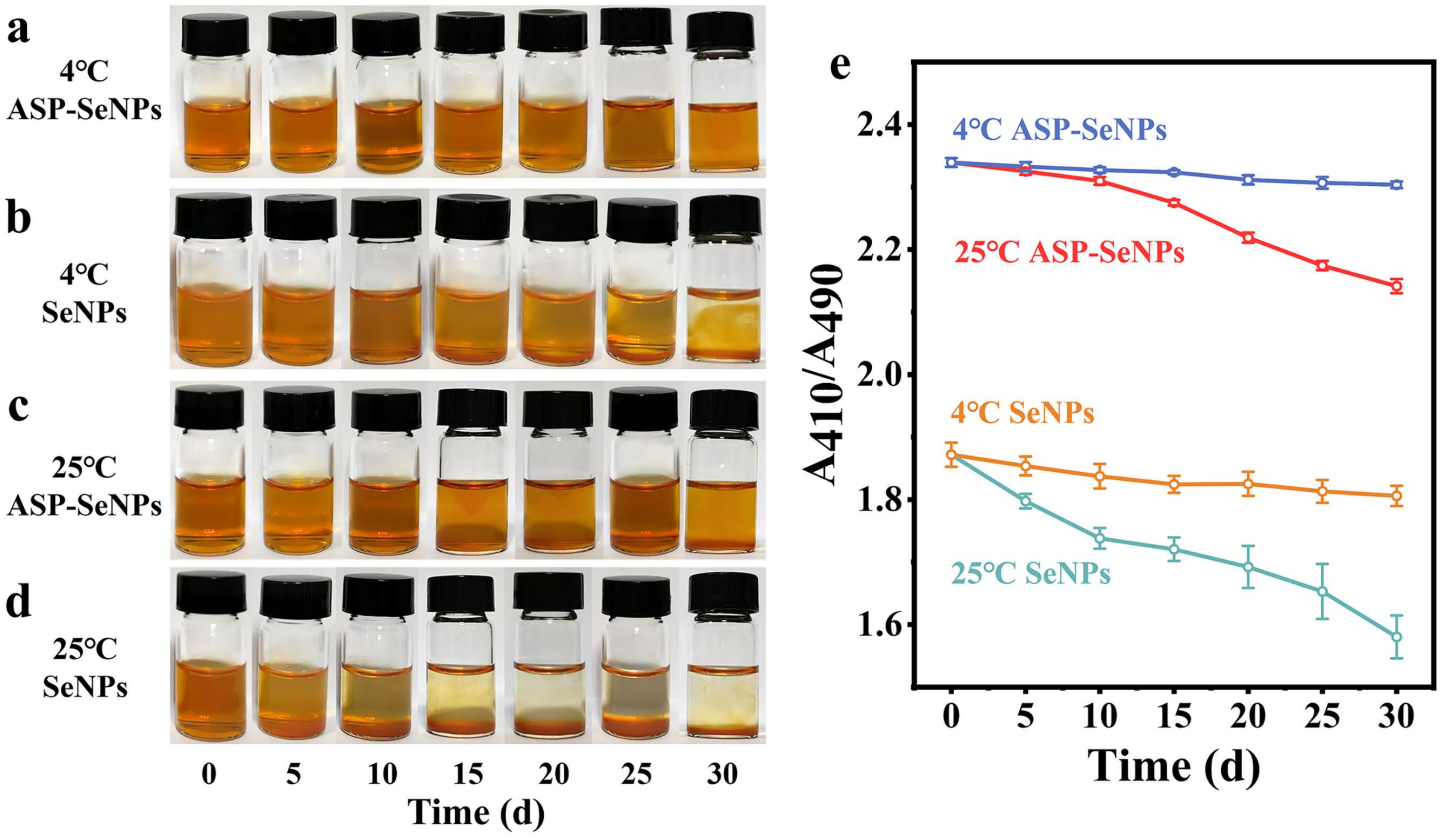
Photographs of ASP-SeNPs and SeNPs left for 0 d-30 d at 4 °C **(a) (b)** and 25 °C **(c,d)** in the dark; Changes in the A410/A490 of ASP-SeNPs and SeNPs left for 0 d-30 d at 4 and 25 °C in the dark.

Since nanoselenium tends to aggregate and since aggregated nanoselenium loses its biological activity, the stability of nanoselenium is one of the key factors determining its application value (35). To assess the stability of SeNPs and ASP-SeNPs, they were stored separately at 4 °C and 25 °C under dark conditions for 30 days. During this period, samples were retrieved every 5 days for observation and color recording, and the changes in A_410_/A_490_ were measured (Figure 8e). As shown in Figure 8a, under dark storage conditions at 4 °C, the color of the ASP-SeNP solution remained stable without aggregation, and A_410_/A_490_ gradually stabilized over time. Moreover, as shown in Figure 8b, under the same storage conditions at 4 °C in the dark, the color of the SeNP solution was unstable, with aggregation occurring by Day 15 and precipitation becoming highly evident by Day 30. Particle aggregation in the liquid was visible, and the A_410_/A_490_ ratio continued to decrease over time, indicating a gradual decline in SeNP stability.

As shown in Figure 8c, the color of the ASP-SeNPs stored in the dark at 25 °C initially remained relatively stable. However, as the storage time increased, the solution became turbid, and by 30 days, a small amount of precipitation was observed. The A_410_/A_490_ of ASP-SeNPs decreased gradually over time, with a smaller decrease than that of the SeNPs under the same storage conditions. However, the decrease was greater than that of ASP-SeNPs stored at 4 °C. As shown in Figure 8d, the solution of SeNPs stored at 25 °C in the dark became turbid after 10 days, and precipitation was observed after 15 days. The A_410_/A_490_ also decreased rapidly, further indicating the extreme instability of SeNPs.

Previous studies have confirmed that the structure of polysaccharides plays an important role in improving the particle size and stability of SeNPs. When polysaccharides are present in the system, the stability of the solution is much greater than when polysaccharides are not present (36). This is because the instability of nanoparticles primarily stems from agglomeration caused by van der Waals forces, while the presence of ASP increases the effective distance between particles, preventing them from approaching within the range where van der Waals forces become dominant. Temperature is also one of the factors affecting the stability of a solution. Compared with those at refrigeration, the rates of molecular movement and interaction increase at room temperature, which accelerates SeNP aggregation (37). Moreover, when the ambient temperature increases, the viscosity of polysaccharides decreases, and the surface charge of SeNPs weakens, thereby reducing the stability of the solution (38). However, ASP-SeNPs exhibit a higher absolute zeta potential than SeNPs. When two particles carrying the same charge approach each other, a strong electrostatic repulsive force is generated. Therefore, regardless of temperature, ASP-SeNPs consistently demonstrate superior stability compared to SeNPs.

The results of the stability tests indicate that ASP-SeNPs, prepared using ASP as a dispersant and stabilizer, exhibit greater stability than traditional SeNPs. These solutions remained stable when stored at 4 °C in the dark for 30 days. Even when stored at room temperature (25 °C) in the dark, they maintain a relatively stable state for approximately 20 days.

### 3.9 Results and analysis of *in vitro* antioxidant activity

#### 3.9.1 Analysis of DPPH radical scavenging assay results

**Figure 9:**
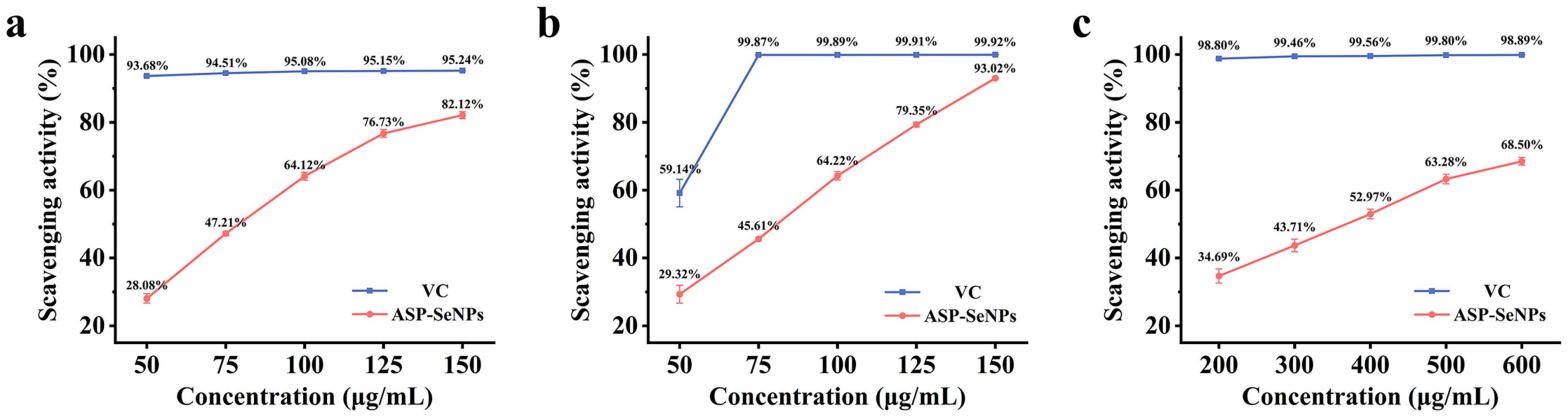
DPPH **(a)**, ABTS **(b)**, and hydroxyl **(c)** radical scavenging abilities of ASP-SeNPs and Vc.

DPPH (1,1-diphenyl-2-picrylhydrazyl) is a stable radical that can be neutralized by antioxidants. Therefore, the DPPH radical scavenging assay can be used to investigate the antioxidant activity of ASP-SeNPs, providing important reference data for subsequent related studies (39). As shown in Figure 9a, when VC was used as the control, within the concentration range of 50–150 μg/mL, the scavenging rate of ASP-SeNPs for DPPH tended to increase. When the concentration reached 150 μg/mL, the DPPH scavenging rate reached 82.12%. Calculations revealed that the IC_50_ of ASP-SeNPs was 76.789 μg/mL.

#### 3.9.2 Analysis of ABTS radical scavenging assay results

Since ABTS radicals can be converted into nonradicals by accepting electrons from antioxidants, the ABTS radical scavenging assay is commonly used to assess the antioxidant activity of samples (40). As shown in Figure 9b, within the concentration range of 50–150 μg/mL, the ASP-SeNPs exhibited significant scavenging activity against ABTS radicals in a concentration-dependent manner. At a concentration of 150 μg/mL, the ABTS radical scavenging rate reached 93.02%, which was very close to the scavenging rate of VC at the same concentration. After the data were analyzed, the IC_50_ of ASP-SeNPs was calculated to be 74.927 μg/mL.

#### 3.9.3 Analysis of hydroxyl radical scavenging assay results

The Fenton reaction can generate hydroxyl radicals. When salicylic acid was added to the reaction system, the generated hydroxyl radicals reacted with salicylic acid to form 2,3-dihydroxybenzoic acid, which exhibited a characteristic absorption peak at 510 nm. If a test substance with hydroxyl radical scavenging activity is added to the reaction system, the amount of hydroxyl radicals generated decreases, thereby reducing the formation of colored compounds. At this point, measuring the absorbance of the reaction mixture containing the test substance at 510 nm can reflect the antioxidant properties of the sample mixture (41). As shown in Figure 9c, when the scavenging rate of vitamin C at the same concentration was used as a control, within the concentration range of 200–600 μg/mL, the hydroxyl radical scavenging rate of the ASP-SeNPs increased with increasing concentration, but the increase was smaller than that of the two aforementioned *in vitro* antioxidant assays. At a concentration of 600 μg/mL, the hydroxyl radical scavenging rate was 68.50%. The calculations yielded an IC_50_ value of 343.419 μg/mL for the ASP-SeNPs.

## 4 Conclusion

This study utilized ASP as a stabilizer to prepare a novel type of nano-selenium particle with controllable size and higher safety. Under the optimized preparation conditions obtained through response surface experiments, the synthesized ASP-SeNPs exhibited a relatively uniform spherical dispersion, with a surface elemental composition consisting primarily of C (40.33%), O (11.00%), and Se (48.67%), with a particle size distribution ranging from 58 to 123 nm and an average potential of −27.8 mV. The characterization results revealed that the binding between ASP and SeNPs occurred primarily via hydrogen bonds or coordination bonds without altering the structures of either component. ASP-SeNPs exhibit superior colloidal stability compared to conventional SeNPs, remaining stable after 30 days of storage at 4 °C under light-protected conditions., and they can maintain a relatively stable state for up to 20 days even at room temperature (25 °C). *In vitro* antioxidant test results revealed that the ASP-SeNPs exhibited good scavenging ability against DPPH radicals, ABTS radicals, and hydroxyl radicals.

In summary, as a green-synthesized biomolecule with excellent activity, ASP-SeNPs hold great potential in improving selenium deficiency and maintaining bodily health.

## Data Availability

The original contributions presented in the study are included in the article/Supplementary material, further inquiries can be directed to the corresponding authors.
