# Supplementary material for: Preparation and characterization of Acanthopanax polysaccharides nanoselenium with enhanced stability and antioxidant activity
Source: Front Nutr. 2025 Nov 19;12:1712826. doi: 10.3389/fnut.2025.1712826 (PMC12672261; doi:10.3389/fnut.2025.1712826)
Supplement: Supplementary file 1 [file Table_1.docx]

**Table S1** Plackett–Burman experiment design and results

| No. | A  mmol/L | B | C  % | D  °C | E  min | F  mg/mL | A410/A490 |
| --- | --- | --- | --- | --- | --- | --- | --- |
| 1 | 5 | 3:1 | 50 | 50 | 80 | 4 | 2.010883 |
| 2 | 5 | 1:1 | 30 | 50 | 160 | 4 | 1.984331 |
| 3 | 5 | 1:1 | 50 | 70 | 160 | 4 | 1.927677 |
| 4 | 5 | 1:1 | 50 | 70 | 80 | 6 | 1.994746 |
| 5 | 3 | 3:1 | 50 | 50 | 160 | 6 | 2.079939 |
| 6 | 3 | 1:1 | 30 | 50 | 80 | 4 | 2.019236 |
| 7 | 3 | 1:1 | 50 | 50 | 160 | 6 | 2.074977 |
| 8 | 3 | 3:1 | 30 | 70 | 160 | 4 | 2.021647 |
| 9 | 3 | 3:1 | 50 | 70 | 80 | 4 | 2.044685 |
| 10 | 5 | 3:1 | 30 | 50 | 80 | 6 | 2.057271 |
| 11 | 3 | 1:1 | 30 | 70 | 80 | 6 | 2.085921 |
| 12 | 5 | 3:1 | 30 | 70 | 160 | 6 | 2.054122 |

**Table S2** Box–Behnken experiment design and results

| No. | A  (mmol/L) | B | C  (mg/mL) | A410/A490 |
| --- | --- | --- | --- | --- |
| 1 | 4.4 | 2.8:1 | 3.8 | 2.205869602 |
| 2 | 4 | 3:1 | 3.8 | 2.192554302 |
| 3 | 4 | 2.8:1 | 3.6 | 2.189870126 |
| 4 | 4.4 | 2.6:1 | 3.6 | 2.181682115 |
| 5 | 4.4 | 3:1 | 4 | 2.194829193 |
| 6 | 4 | 2.8:1 | 4 | 2.195683083 |
| 7 | 4.4 | 2.8:1 | 3.8 | 2.204247887 |
| 8 | 4.8 | 3:1 | 3.8 | 2.189530857 |
| 9 | 4.4 | 3:1 | 3.6 | 2.190105582 |
| 10 | 4.8 | 2.8:1 | 3.6 | 2.180011586 |
| 11 | 4.4 | 2.6:1 | 4 | 2.187068491 |
| 12 | 4 | 2.6:1 | 3.8 | 2.186411402 |
| 13 | 4.8 | 2.8:1 | 4 | 2.204016836 |
| 14 | 4.4 | 2.8:1 | 3.8 | 2.204218468 |
| 15 | 4.4 | 2.8:1 | 3.8 | 2.204332082 |
| 16 | 4.4 | 2.8:1 | 3.8 | 2.194452492 |
| 17 | 4.8 | 2.6:1 | 3.8 | 2.186596092 |
